# A covered eye fails to follow an object moving in depth

**DOI:** 10.1038/s41598-021-90371-8

**Published:** 2021-05-26

**Authors:** Arvind Chandna, Jeremy Badler, Devashish Singh, Scott Watamaniuk, Stephen Heinen

**Affiliations:** 1 The Smith-Kettlewell Eye Research Institute, San Francisco, CA USA; 2 Department of Sensory and Sensorimotor Systems, Max Planck Institute of Biological Cybernetics, Tübingen, Germany; 3 Department of Psychology, Wright State University, Dayton, OH USA

**Keywords:** Smooth pursuit, Object vision

## Abstract

To clearly view approaching objects, the eyes rotate inward (vergence), and the intraocular lenses focus (accommodation). Current ocular control models assume both eyes are driven by unitary vergence and unitary accommodation commands that causally interact. The models typically describe discrete gaze shifts to non-accommodative targets performed under laboratory conditions. We probe these unitary signals using a physical stimulus moving in depth on the midline while recording vergence and accommodation simultaneously from both eyes in normal observers. Using monocular viewing, retinal disparity is removed, leaving only monocular cues for interpreting the object’s motion in depth. The viewing eye always followed the target’s motion. However, the occluded eye did not follow the target, and surprisingly, rotated out of phase with it. In contrast, accommodation in both eyes was synchronized with the target under monocular viewing. The results challenge existing unitary vergence command theories, and causal accommodation-vergence linkage.

## Introduction

Two neural systems are postulated to move the eyes together; a conjugate system that shifts gaze between objects on the fronto-parallel plane, and a vergence system that shifts gaze between objects at different depths^2^. Single commands from each system are thought to sum to rotate the eyes to view any location^3–12^. The conjugate command innervates complementary extraocular muscles, e.g., the medial rectus of one eye and the lateral rectus of the other, to rotate the eyes in the same direction^2^. The vergence command conversely innervates the same muscle groups, e.g., both medial recti, to rotate the eyes in opposite directions as mirror images^13–17^. For both conjugate and vergence eye movements, inhibitory commands relax opposing muscles in each eye^18^.

Classic models of the normal mature binocular system treat the conjugate^19,20^ and the vergence^21^ systems as largely independent^22,23^ and their single commands are maintained until the motor nuclei. The models also posit that all eye movements are a response to retinal signals, but that vergence is driven additionally by monocular depth cues and, critically, by a unitary accommodation signal that focuses the intraocular lens to clearly view objects in depth^24–27^. Binocular coordination is fragile however, and abnormal visuomotor development often leads to strabismus in which the eyes are misaligned^28–30^. Strabismus is often accompanied by amblyopia, manifest as poor acuity in the misaligned eye^31–34^. Many strabismus subtypes are believed to result from dysfunctional cross-links between vergence and accommodation^35–37^. Vergence and accommodation models are important because they guide surgical and optical management of most types of primary childhood strabismus^38,39^.

There are several potential problems with previous studies that were used to construct these models. The earliest models^14,24,40–43^ were derived using binocular viewing, when similar accommodation and vergence demands were placed on both eyes. This method makes assessing the unitary nature of vergence, accommodation and the strength of accommodation-vergence links difficult because the behavior of both eyes and the accommodative state are correlated with the target’s location. Breaking the correlation with target position by providing unequal inputs to the two eyes provides a better test of whether a unitary command drives both eyes, and whether accommodation and vergence are causally linked. Some studies measured behavior of the viewing eye with one eye occluded, but within dichoptic viewing paradigms^27,44,45^. However, this method usually eliminates monocular depth cues that are ubiquitous in natural environments and drive accommodation and vergence^46–52^. Furthermore, the use of non-accommodative targets^48,53–56^ and the absence of peripheral fusional cues^51,52,57,58^ in previous studies prevented the accommodation and vergence systems from operating naturally. Finally, the classic method of calculating vergence (Eq. 1) and version (Eq. 2) obscures the individual eye rotations because they result in a single value that could be obtained from an infinite number of combinations of right eye and left eye rotations.

1 $${\text{Vergence }} = {\text{ RE }}{-}{\text{ LE}},$$

2 $${\text{Version }} = \, \left( {{\text{RE }} + {\text{ LE}}} \right)/{2},$$

where *RE* is the right eye position, and *LE* is the left eye position.

A previous study^59^ investigated smooth pursuit of target motion in depth in monkeys. While these researchers found evidence of asymmetric vergence during binocular viewing in support of independent control, target motion in their study was usually aligned with one eye, where asymmetric vergence is expected.

Here we investigate in humans eye movements and accommodation while the eyes smoothly pursue a physical target moving in depth on the midline. During binocular viewing in this situation, the eyes rotate symmetrically in opposite directions in accordance with Hering’s law, and are modulated by accommodation^2,25,26^. The target moved in a free-field open laboratory environment preserving monocular depth and peripheral fusional cues. Unlike most previous studies testing gaze shifts in depth between static targets, our target moved continuously throughout each trial. Our primary manipulation was covering an eye, a situation in which both Hering’s law and models of accommodation and vergence predict that the eyes should continue to converge as they do during binocular viewing.

## Results

**Figure 1:**
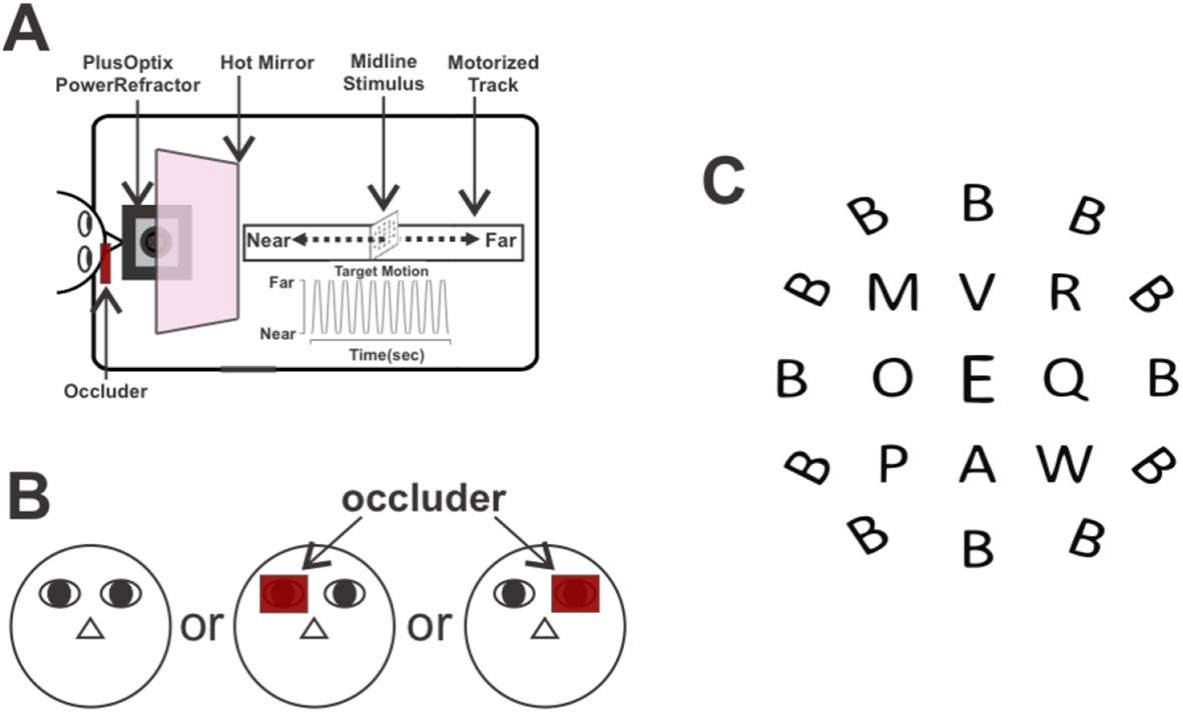
Experimental paradigm. **(A)** The target moved back and forth on the midline (33 cm–67 cm from observer) for 63 s at 30 cm/s with an acceleration of 50 cm/s^2^ on a motorized track while accommodation and vergence were recorded. **(B)** Viewing was either binocular or monocular with the left eye or the right eye covered with an IR-passable occluder. **(C)** Observers fixated the central "E" of the letter matrix stimulus.

We recorded eye rotation and accommodation (refraction) from both eyes simultaneously during both binocular and monocular viewing from 14 normal participants (11 female, age 15–49). All had normal or corrected to normal visual acuity, normal oculomotor and sensory status, and no ocular pathology as assessed with standard clinical tests (Supplementary table S1). Participants viewed a printed matrix of letters mounted on a post and were instructed to look at the central optotype (“E”) to ensure appropriate accommodative effort (Fig. 1A,C). The target moved forward and backward in depth (10 cycles) on a motorized track positioned perpendicular to and aligned with the midline of the participant’s head stabilized with a chinrest^60–62^. Viewing was either binocular, or monocular with one eye occluded by an IR-passible filter that blocked vision but allowed eye movement and accommodation measurement (Fig. 1B).

**Figure 2:**
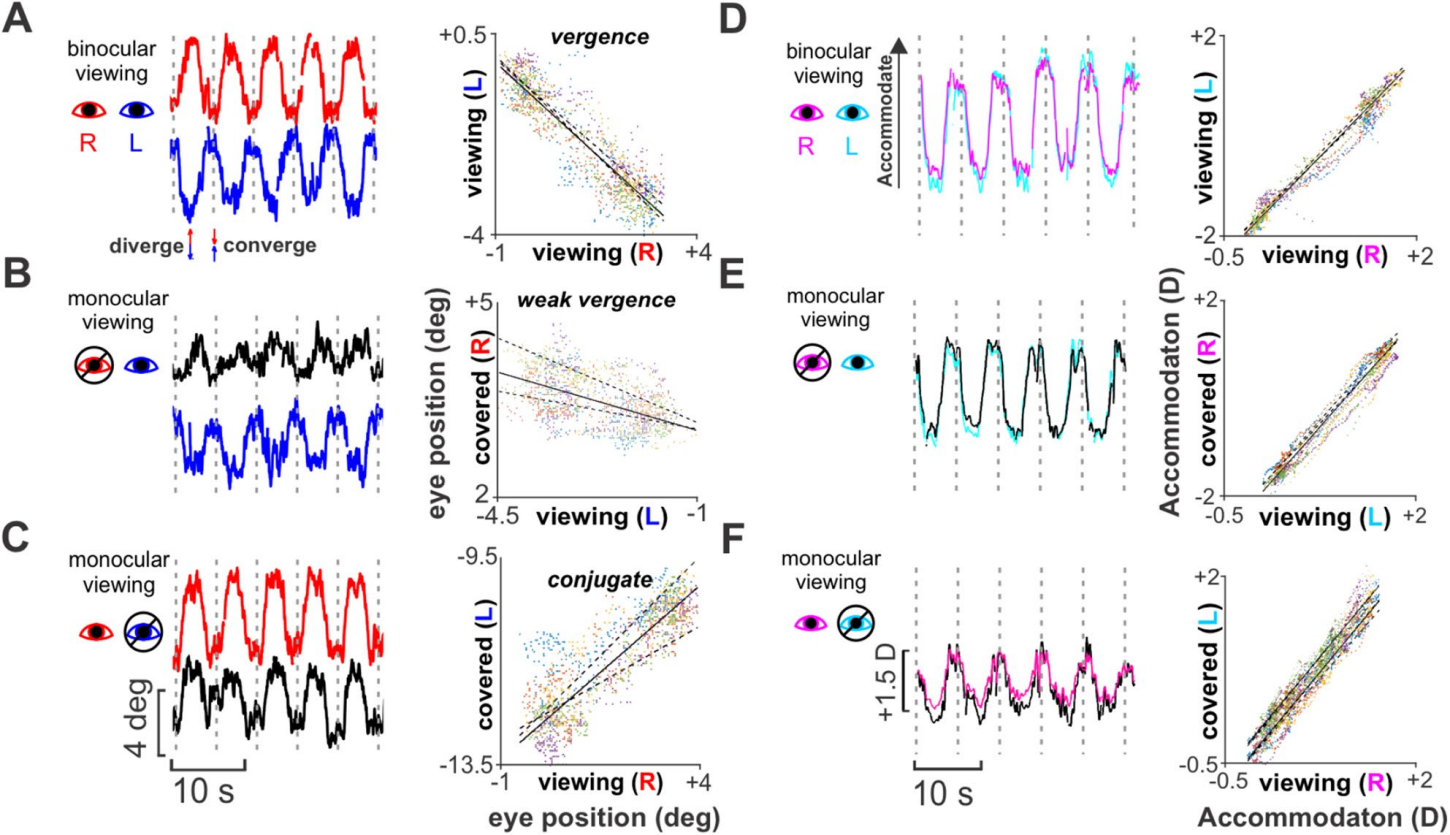
Vergence and accommodation with binocular and monocular viewing. **(A)** Eye position traces and gaze correlation plots showing interocular gaze slope (IGS) for S13 during binocular and **(B )** monocular viewing. **(C)** Eye position traces and gaze correlation plots measured during monocular viewing for another subject (S11). **(D–F)** Corresponding accommodation traces and accommodation correlations (interocular accommodation slope, lAS) for **(A–C)**. Dot colors on correlation plots correspond to different cycles of target movement, and dotted lines of fit correspond to 95% confidence intervals of regression. All gaze and accommodation traces are separated into cycles using vertical dotted lines.

During binocular viewing, participants made vergence eye movements appropriate to follow the midline target. Figure 2A shows raw traces from both eyes of one observer (S13) pursuing five cycles of target motion. The eyes’ traces are counter-phased in time, indicating that they rotated in opposite directions appropriate for midline vergence. We characterized the relative eye rotations by using a linear regression to compute the interocular gaze slope (IGS) for eye position at every time point (see Methods). Vergence pursuit resulted in an almost perfect negative correlation (IGS = − 0.92), as it should (Fig. 2A, right).

However, during monocular viewing, while the viewing eye continued moving as during binocular viewing, the occluded eye unexpectedly rotated inappropriately for the midline target’s motion in depth. In some cases the rotations were vergence-like, directed opposite those of the viewing eye, but with reduced amplitude (Fig. 2B). Reflecting this, the eye position correlation was negative, but with a greatly reduced slope (IGS = -0.25; Fig. 2B right). Note that asymmetric vergence has been observed in the past for off-center gaze shifts in depth (e.g.^4,59^), but never documented with continuous midline target motion that specifies symmetric ocular behavior. In other cases of monocular viewing, the rotation of the occluded eye was even more surprising, as it turned in the *same* direction as the viewing eye, with nearly the same magnitude. We show this conjugate-like behavior (version) in a different observer (S11) in Fig. 2C. Here, the eye position correlation is positive (IGS = 0.78) (Fig. 2C, right). It was not possible to predict one covered eye’s behavior from the other, and in most observers, the covered eyes’ behaviors were different. Version has not previously been reported in normal observers viewing targets specifying a midline vergence gaze shift. We confirmed these results with a high-resolution EyeLink 1000 eye tracker (supplementary Figure S1).

In prevailing models, accommodation drives vergence and vice versa. We found that during binocular viewing, the eyes obeyed the models in that symmetric vergence (Fig. 2A) was accompanied by symmetric accommodation (Fig. 2D), i.e., when the eyes converged and diverged, accommodation increased and decreased proportionately, and in phase with vergence. The interocular accommodation slope (IAS; see Methods) was accordingly close to unity (IAS = 0.99; Fig. 2D). During monocular viewing, accommodation was also symmetrical in the covered and viewing eyes. In Fig. 2E it can be seen that symmetrical accommodation accompanied weak vergence (IAS = 1.07). In Fig. 2F accommodation remains symmetrical even with version rotations (IAS = 1.24).

**Figure 3:**
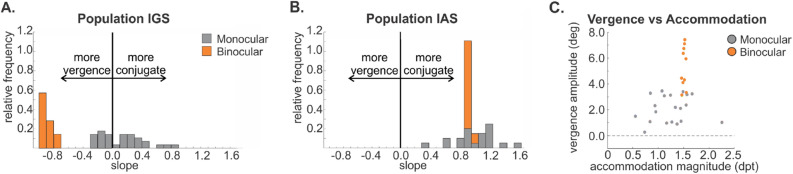
Gaze and accommodation slope summary. **(A)** Stacked frequency distribution of the interocular gaze slopes (IGS). Binocular and monocular viewing conditions are completely dissociated, forming two non-overlapping distributions of IGS. Black vertical line indicates zero slope. **(B)** Stacked frequency distribution of the interocular accommodation slopes (lAS). Binocular and monocular viewing conditions have completely overlapping distributions, showing no asymmetry. **(C)** Classically computed vergence amplitude (Eq. 1) plotted against accommodation magnitude measured in the viewing eye(s).The lack of correlation in both monocular and binocular data refute a causal link between vergence and accommodation in our experiment.

Figure 3 shows population data as stacked histograms for the interocular gaze and accommodation slopes for each eye in all observers. There is no overlap between binocular and monocular viewing for IGS (Fig. 3A). For binocular viewing, the IGS data are distinctly clustered in the vergence range (median = − 0.91; interquartile range = − 0.93 to − 0.85), where perfect vergence IGS is − 1.0. However, for monocular viewing, the IGS values are centered around 0.2 with the majority of covered eyes being directed in the same direction as the viewing eye (median = 0.29; interquartile range = 0.17 to 0.47) and a smaller number showing weak vergence (median = − 0.13; interquartile range = − 0.20 to − 0.04). On the other hand, IASs for the binocular and monocular conditions completely overlap (Fig. 3B; binocular median = 0.97; binocular interquartile range = 0.96 to 0.99; monocular median: 1.09; monocular interquartile range = 0.92 to 1.26) as evidenced by a non-parametric Sign Test (*p* = 0.09).

Finally, if accommodation is driving vergence through the postulated cross-link^26^, larger accommodation magnitudes should yield more negative IGS values. To test this, we assessed the correlation between the accommodation magnitude in the viewing eye and the computed IGS. The correlation was weak (Kendall correlation = − 0.19, p = 0.04) as would be expected given the almost complete dissociation between accommodation and vergence in our population (Fig. 3A,B). Our IGS measure is novel and has not been previously used to assess the degree to which accommodation drives vergence. To validate our method, we computed vergence magnitudes using the classic method (Eq. 1) for each observer and correlated these with accommodation in the viewing eye (Fig. 3C). This correlation was even weaker (Kendall correlation = − 0.02, p = 0.75) than that obtained with IGS. We also correlated vergence magnitudes with accommodation during binocular viewing, which based on the models should show a perfect correlation. Critically, despite virtually veridical vergence in the binocular condition, the magnitude of accommodation spanned a wide range resulting in a weak though significant correlation (Kendall correlation = 0.28, p = 0.0003). Overall, consistent with past research^63–65^,we found that accommodation during monocular viewing was lower than during binocular viewing (Wilcoxon Rank Sum = 0.0015, p = 5.52 × 10^–8^).

The dissociation between vergence and accommodation in our paradigm suggests that accommodation and other monocular cues drives vergence only weakly, if at all. Furthermore, our finding that the covered eye did not rotate appropriately for target motion on the midline lead us to doubt that a unitary command was driving vergence, as the classical theory of binocular eye movement control states. But the mixed behavior of the covered eye we observed can be described by a mathematical sum of vergence and conjugate commands according to the classical theory^2^. This theory can not only describe ocular behavior in our experiments, but it has sufficient free parameters to describe any observed pair of eye rotations (“a mathematical tautology”^52^), rendering it untestable and unable to suggest a mechanism for binocular eye movement control.

**Figure 4:**
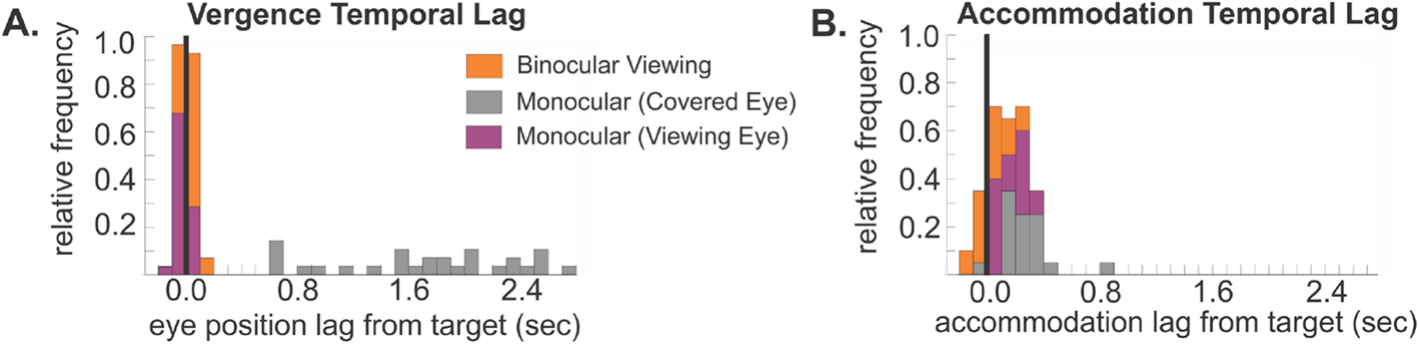
Vergence and accommodation temporal lag. **(A)** Stacked frequency distribution of the timing difference between vergence and target. **(B)** Stacked frequency distribution of the timing difference between accommodation and target. Black vertical line indicates zero temporal lag.

Strong evidence against a unitary vergence command can be gleaned from the timing of the covered eyes’ rotations, as they appeared delayed relative to the target. We quantified this with a cross-correlation analysis (see Methods). While the viewing eye remained largely in temporal alignment with the target (median delay = − 0.02 s; interquartile range = − 0.03 s to 0.0 s), the covered eye was significantly delayed (median delay = 1.74 s; interquartile range = 1.23 s to 2.58 s); Friedman with Tukey–Kramer post hoc, *p* < 0.01) (Fig. 4A). In the classic theory, the signals for vergence and version are in antiphase and sum to produce the final eye movement. However, our stimulus moved sinusoidally and it is impossible to get a phase shift by summing two sinusoids of equal frequency that are 180° out of phase. Thus the classic theory cannot explain the observed temporal delays between the viewing and covered eyes’ movements. Although vergence temporal lags for periodic targets have been reported previously in normal observers^26,66^, there are no prior reports of different time lags for the left and right eyes while responding to the same target.

We also analyzed the timing of the accommodation responses (Fig. 4B). While there was more temporal lag for accommodation overall during monocular compared to binocular viewing, there was no significant difference between the lag values of the covered and viewing eye under monocular conditions (Friedman with Tukey–Kramer post hoc, *p* > 0.01). The rotation of each covered eye lagging the target, combined with the presence of synchronized accommodation between the covered and viewing eye is further evidence that a causal link between the accommodation and vergence systems is absent or weak when an eye is occluded.

**Figure 5:**
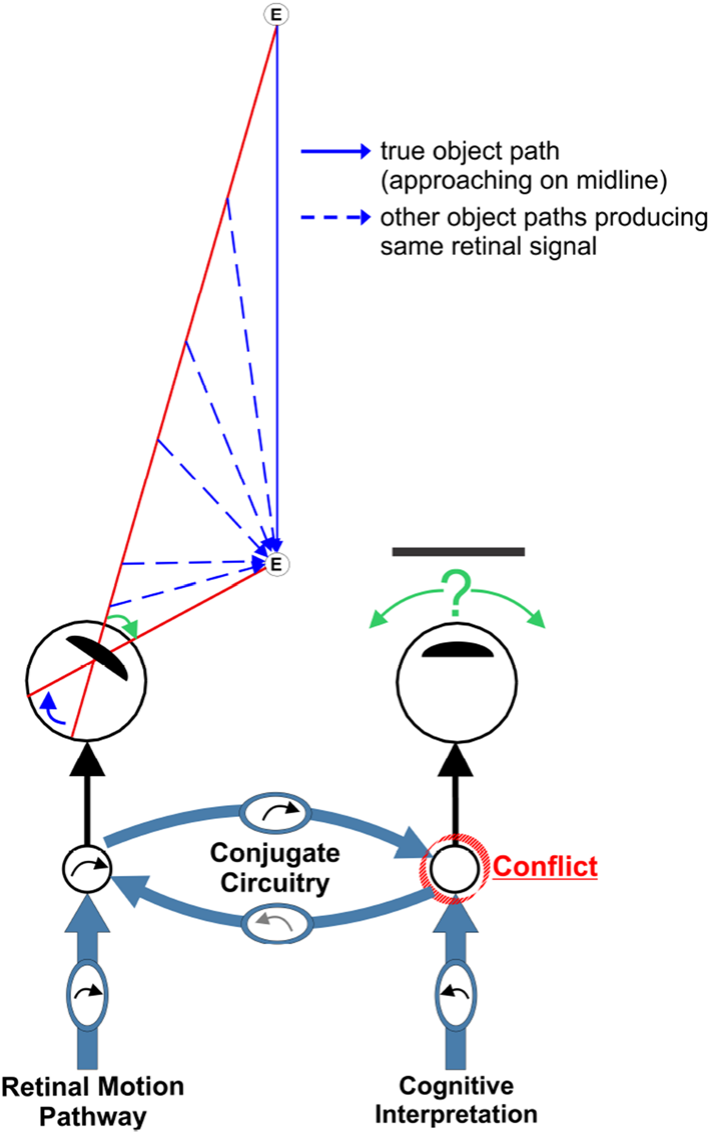
Controlling occluded eye rotations. Retinal motion in the viewing eye is ambiguous and the target's depth component is interpreted from monocular depth cues. A vergence command is issued to the covered eye, which conflicts with a conjugate command issued from the viewing eye. Resolution of the conflict determines covered eye rotation.

The ~ 1–2 s delay between the target and rotation of the covered eye, with virtually no delay in the viewing eye, is inconsistent with a single vergence command, or a simple sum of vergence and conjugate commands driving both eyes^2^. We propose instead that neural systems controlling vergence eye movements send independent commands to the eyes as hypothesized previously^22,67^, but instead of ocular control being entirely independent, these commands modulate conjugate circuitry (Fig. 5).

## Discussion

In our scheme (see Fig. 5), the viewing left eye moves appropriately simply by responding to retinal motion. That motion could be produced by an infinite number of target trajectories, all of which would be followed using the same eye rotation. For the covered eye to move appropriately, the brain must interpret the target’s depth trajectory from monocular depth cues such as changing retinal size, occlusion, texture, and perspective^68^, and generate a vergence movement to follow the interpreted motion of the target. However, in parallel, the retinal motion in the viewing eye activates well-established conjugate brainstem circuitry, possibly the same as that used by the vestibulo-ocular reflex VOR^69^. The conjugate circuitry issues a command to the covered eye that conflicts with the command to follow the interpreted object motion. The resolution of the conflict determines the covered eye’s rotation, i.e., if the conjugate signal dominates, a version movement results, while if the vergence signal dominates, vergence results. When both eyes view the target, the conjugate system is either actively suppressed, or because rightward and leftward commands are issued simultaneously these commands then activate opposing conjugate commands that cancel.

A previous study investigated pursuit of a target moving in depth with an occluded eye in a single monkey^59^. Curiously, the behavior of the occluded eye was not quantified but merely described in a footnote. The occluded eye was described as eventually moving in the same direction as the viewing one. It appears this result is consistent with eye movements we observe in the occluded eye in some of our observers, although in their experiment the target motion was displaced from the midline, and not on it as ours was. These results were explained within the context of an independent control model. While it might be possible to explain our results with an independent control model, why independent control would make the covered eye move in the same direction as the viewing eye is unknown. Given the occluded eye has no visual input, independent control should keep that eye fixed. King and Zhou’s explanation of why an occluded eye moved in the same direction as the viewing one was that its movement was influenced by past experience. Past experience does not account for the version we observe in the covered eyes of our human subjects, as their immediately preceding behavior was pursuing midline motion with both eyes viewing, where the eyes rotated oppositely. Past experience also does not account for the monkey’s occluded eye eventually moving in the same direction as the viewing eye, when the monkey previously viewed a target aligned with the viewing eye^59^. Our model suggests that a conjugate component interacts with independent circuitry. Supporting this, several studies found neurons in the conjugate pathway that showed evidence of independent control, while some neurons in these structures carry a conjugate signal^1,70,71^. We think these results are not inconsistent with our model because the gains on the interacting components could vary between subjects, explaining the range of behaviors we see in different occluded eyes in both humans and monkeys.

Our results also challenge models in which accommodation drives vergence and vergence drives accommodation via a causal linkage^25,45^. Because our stimuli are physical targets that move in depth, they have all of the relevant monocular cues for driving the accommodation-driven vergence link, including blur and looming. Our data show that despite a cue-rich environment, vergence during monocular viewing is crippled or absent in the covered eye, yet accommodation continues to function as it does during binocular viewing. Our result is inconsistent with accommodation causally driving vergence during monocular viewing.

Our results suggest that vergence is mediated by sluggish monocular commands originating in the cortex, possibly in the frontal eye fields where neurons active during vergence exist^17^. This command appears to inhibit default conjugate circuitry in the brainstem with varying degrees of success. This conjugate circuitry could involve a subset of neurons in the abducens nucleus of the brainstem which control conjugate eye muscles in both eyes via the 6^th^ cranial nerve that remain active during monocular gaze shifts^1^. During binocular viewing, the activity of these neurons could be overridden by visual signals that drive monocular motor neurons^1^. When one eye is covered, the input to its monocular motor neurons is disabled, allowing the conjugate abducens neurons to control the covered eye.

Our results have significance for understanding childhood strabismus which afflicts as much as 5% of the population^31,32,72^. Strabismus is often predated by poorly controlled binocular alignment followed by persistent misalignment. Strabismus commonly leads to cortical suppression of the deviated eye to avoid double vision, resulting in a monocular-viewing condition mimicked by our paradigm. Knowing the extent to which binocular control in a strabismus patient is dominated by brainstem conjugate circuitry or cortical control could help inform treatment in the clinic.

A dysfunctional relationship between accommodation and vergence is thought to exist in strabismus^73^. In the clinic this relationship is characterized by the ratio between the change in vergence induced by increasing accommodation with lenses and accommodation (AC/A ratio)^74–76^. The AC/A ratio relies on a theoretical causal link between accommodation and vergence^73,77–83^, which our results make tenuous. Thus using the AC/A ratio to help determine the appropriate surgical or optical interventions for strabismus may contribute to their often unpredictable outcomes^55,78,83–94^. Furthermore, our results suggest there may be subtypes of strabismus that arise from primary vergence or accommodation disorders. This may influence whether therapy involves surgery or optical correction. In addition, regularly measuring the behavior of a covered eye in strabismus patients may provide a systematic indicator of disease progression that may be more sensitive than standard clinical tests.

### Significance

Models of ocular control posit that a single command drives vergence, and that accommodation and vergence are causally linked. Our results challenge these prevailing models and support the alternative theory of monocular control^1^. These findings not only suggest a reformulation of oculomotor control mechanisms but also a reassessment of normal oculomotor function, and that of the medical interventions employed when patients present disorders of binocular control such as strabismus.

## Methods

### Subjects

14 participants (3 M, 11F, ages 15–49) were tested (supplementary Table S1). None of the subjects reported vision problems nor had received treatment for clinical eye disorders aside from myopia. Myopic subjects wore their corrective lenses during testing. Most subjects underwent a ~ 30-min clinical exam before testing, where visual acuity, stereoacuity, ocular motility and other characteristics were assessed (see supplementary Table S1); S06 underwent more limited testing. One of the subjects (S01) was a co-author of the study; the rest were naïve as to its design and purpose. Smith-Kettlewell Eye Research Institute’s Institutional Review Board approved all parts of the experiment which was conducted in accordance with the Declaration of Helsinki. All participants provided their informed consent prior to participating in the experiment or had consent provided for them by an informed parent or guardian if under the age of 18. All the authors have no conflicts of interest to report.

### Stimuli

The stimulus was a physical target that moved along a track towards and away from the observer on the midline in a well-illuminated open-laboratory environment, thus preserving all available monocular depth cues. It consisted of a printed array of letters (Fig. 1C), each of which subtended 1.21 visual degrees at 33 cm and 0.34 degrees at 67 cm, to encourage accommodation by fixating on the central letter and keeping it clear. The target array was centered on a post mounted on a movable platform that moved along a custom-built 6 m motorized track fixed to a table. Platform motion was driven by a stepper motor (Phidgets, Inc.) controlled by a computer. Beginning at a distance of 33 cm, the target moved back and forth between 33 and 67 cm with a peak velocity of 30 cm/s and acceleration of 50 cm/s^2^, pausing for 1 s at each endpoint (Fig. 1A), for a total of 10 cycles. Maximum dioptric change velocity is ~ 1.9 dpt/s, well below peak accommodation speeds^95–97^.

### Eye position and accommodation recording

Eye movement data were collected using one of two methods. For all subjects, a PlusOptix Power Refractor III (PlusOptix GmbH, Nuremberg, Germany) measured the dioptric power (accommodation) and gaze position for both eyes simultaneously. The PlusOptix uses photoretinoscopy to continuously measure the near triad^98^. However, it has limited position resolution (0.47°) and a 50 Hz sampling rate. The PlusOptix camera was placed below the table and recorded the subject’s eyes via an aperture and 45° angled hot mirror (Fig. 1A). Subjects were seated with their head in a chinrest behind the mirror.

Four subjects also had gaze position without accommodation recorded at high resolution using an EyeLink 1000 eyetracker. Recordings were again binocular but sampled at 500 Hz, with a spatial resolution of less than 0.01°. The EyeLink tower mount contained its own chinrest, forehead-rest and angled hot mirror, and was modified to record binocularly with an angled camera and single active LED bank.

### Alignment

The chinrest was aligned to the track midline before each experiment using a laser sight. Observers were aligned to the midline using the chinrest. A video camera mounted overhead recorded each data session to verify that alignment was maintained. To confirm repeatability, a subset of the observers remained in the chinrest during three consecutive data collection blocks (binocular viewing, 1st eye viewing, 2nd eye viewing), then exited and returned for three additional blocks in reverse order. Alignment for a subset of participants was verified using overhead photographs.

### Occluders

The occluders were infrared-passable filters that block visible light (cutoff ~ 750 nm; ePlastics, San Diego, CA). They appeared completely opaque to the observer other than a faint image of the eye tracker light sources, which did not interfere with eye movement recording. Tests using an artificial eye (Optical Retinoscope Schematic Model Eye Practice Metal Model Eye Teaching Practice Eye, Huanyu Instrument) showed the occluders added a maximum of ± 0.05 dpt additional noise (mean = − 0.0453, SD = 0.0121, rms = 0.7148) to the measured accommodation data. The occluder was mounted on the chinrest and remained in place during the entirety of a monocular viewing block.

### Calibration

The purpose of calibration was to standardize gaze position and accommodation data across subjects, to enable inter-subject comparisons, while preserving differences between viewing conditions for a single subject. Binocular eye-tracker calibration for targets in multiple depth planes is a nontrivial problem^99^, as vergence errors increase with distance from the calibration plane^100^. We therefore calibrated the eye position data returned by the PlusOptix and EyeLink using a two-stage process. Only horizontal eye movements were characterized.

First, we calculated the average gaze and accommodation values that corresponded to the near and far excursion positions of the eyes. This was calculated as the mean of the upper 67th and lower 33rd percentiles, respectively, of the gaze and accommodation data (with the sign flipped for the right eye since its rotation values are normally negative, i.e. leftward). In other words, at the near-most fixation position, absolute gaze rotation and accommodation are maximal, while at the far-most fixation position they are minimal. We heretofore refer to these averages as Offset_Near_ and Offset_Far_. The average amplitude of the gaze and accommodation shift for each eye was calculated as the absolute difference between the values for the near and far target positions, i.e. abs(Offset_Near_ − Offset_Far_).

Next, the binocular viewing data were scaled to the geometric ideal based on the target position and a population-typical interpupillary distance (IPD) of 60 mm^101^. Therefore, the ideal angular rotation of the left eye (R_L_) at a given distance D is:

3 $${\text{R}}_{{\text{L}}} \left( {\text{D}} \right) \, = {\text{ tan}}^{{ - {1}}} \left( {0.{5}*{\text{IPD }}/{\text{ D}}} \right),$$

where rightward is positive by standard convention. Angular rotations correspond to eyetracker eye position values. Expected rotation of the right eye (R_R_) is simply the inverse of the left:

4 $${\text{R}}_{{\text{R}}} = \, - {\text{R}}_{{\text{L}}} .$$

For the near and far target distances, the ideal rotation values are thus ± 5.4° and ± 2.7° respectively. Eye rotations are related to vergence in meter angles (V) by the following formula:

5 $${\text{V }} = {\text{ c }}*{\text{ abs}}\left( {{\text{R}}_{{\text{R}}} - {\text{ R}}_{{\text{L}}} } \right) \, /{\text{ IPD}},$$

where c is the conversion factor of 1.75 pd/deg. Ideal accommodation amplitudes are simply the inverse of the target distances, minus a conservative 0.5 dpt to account for lag^102,103^, (i.e., 2.5 dpt and 1.0 dpt for the near and far distances respectively). For each of right and left gaze and accommodation, a scaling factor (S) was computed as:

6 $${\text{S }} = {\text{ ideal amplitude }}/{\text{ measured average amplitude}}.$$

For both binocular and monocular gaze data, the final step was to subtract the calculated gaze position baseline (Offset_Near_ or Offset_Far_) to set the minimum value to zero, then multiply by the previous scaling factor. For the monocular data, the difference between the current baseline and the baseline under binocular conditions was added back, to determine the shift relative to binocular conditions (i.e., the phoria).

For a subset of subjects, a 2-D eye position calibration was also performed by measuring eye position while observers sequentially fixated one central and eight peripheral positions on a printed card, at a distance of 33 or 67 cm. Binocular and each monocular viewing condition were calibrated separately. For selected subjects, the PlusOptix accommodation measures were also calibrated for each eye during monocular viewing, by sequentially placing different power lenses (− 2, − 1, 0, + 1, + 2) over the occluded eye^104,105^.

### Data analysis

All analyses were performed using MATLAB (MathWorks, Inc., Natick, MA). Dioptric power and eye position were smoothed using a 200 ms (10 sample) moving average filter, respectively. Blinks were identified and removed manually.

To characterize ocular behavior between the two eyes, we calculated interocular gaze slope (IGS) using a bisquare linear regression (MATLAB’s ‘robustfit’) of covered eye position vs. viewing eye position, or in the binocular case, the average of regressions for left vs. right and right vs. left. The tuning parameter was set to 2.34, one half of the default, which decreased the relative weight of outlying data points from blink artifacts or saccades. The IGS values range from − 1 when the eyes move in opposite phase (i.e. vergence), through zero when they are uncorrelated and + 1 when they are conjugate.

Since data points from the entire trial were used to compute IGS, its variability was quantified using bootstrap statistics. Each trial was composed of ten periods of target motion. Each period was sampled randomly with replacement 1000 times, and slopes were recalculated for each sample. The 95% confidence intervals correspond to values between the 2.5th and 97.5th percentiles of the bootstrap sample distribution.

A cross-correlation analysis was used to assess the temporal displacement between eye and target position for all conditions. Target position was expressed relative to each eye (i.e. a receding target corresponded to a leftward position shift for the left eye but a rightward position shift for the right eye). For accommodation, the target position in diopters was the same for both eyes (decrease for receding target and increase for approaching target). For each cycle of target motion, a cross-correlation vector was computed by temporally shifting the gaze or accommodation data relative to the corresponding target position, in single sample increments, up to ± 4.5 s. The location of the maximum value of that vector corresponds to the time shift where the eye and target traces are maximally correlated. Positive time shifts correspond to eye signal lag; i.e., the eye signal would need to be shifted forward in time by that amount to maximize correspondence with the target signal. Negative time shifts are the reverse, corresponding to eye signal lead. Note that due to the periodicity of the data, three-quarter cycle lag (1.58 s) lead is indistinguishable from a quarter-cycle lag.

For our IGS and IAS metrics, the eyes behaved no differently under binocular viewing (sign test, p > 0.05). Under monocular viewing, right and left eyes behaved no differently if they were the viewing eye, and they also behaved no differently if they were the covered eye (sign test, p > 0.05). As a result, the data from the left and the right eyes was collapsed into a singular data set for further statistical analysis. Comparisons between the aforementioned conditions were done using the Friedman test (Tukey–Kramer post hoc). The use of non-parametric tests was supported by the Shapiro–Wilk test, which revealed that multiple homogenous blocks of data contained both parametric and non-parametric data^106^.

## Supplementary Information

Supplementary Information.
