# Supplementary material for: A covered eye fails to follow an object moving in depth
Source: Sci Rep. 2021 May 26;11:10983. doi: 10.1038/s41598-021-90371-8 (PMC8154899; doi:10.1038/s41598-021-90371-8)
Supplement: Supplementary file 1 — Supplementary Information. [file 41598_2021_90371_MOESM1_ESM.pdf]

A covered eye fails to follow an object moving in depth

Arvind Chandna[1], Jeremy Badler[2], Devashish Singh[1], Scott Watamaniuk[1, 3], Stephen Heinen[1]

[1]The Smith-Kettlewell Eye Research Institute, San Francisco, CA; [2]Max Planck Institute of Biological Cybernetics, Department of Sensory and Sensorimotor Systems, Tübingen, Germany; [3]Department of Psychology, Wright State University, Dayton, Ohio

Supplementary Figure S1

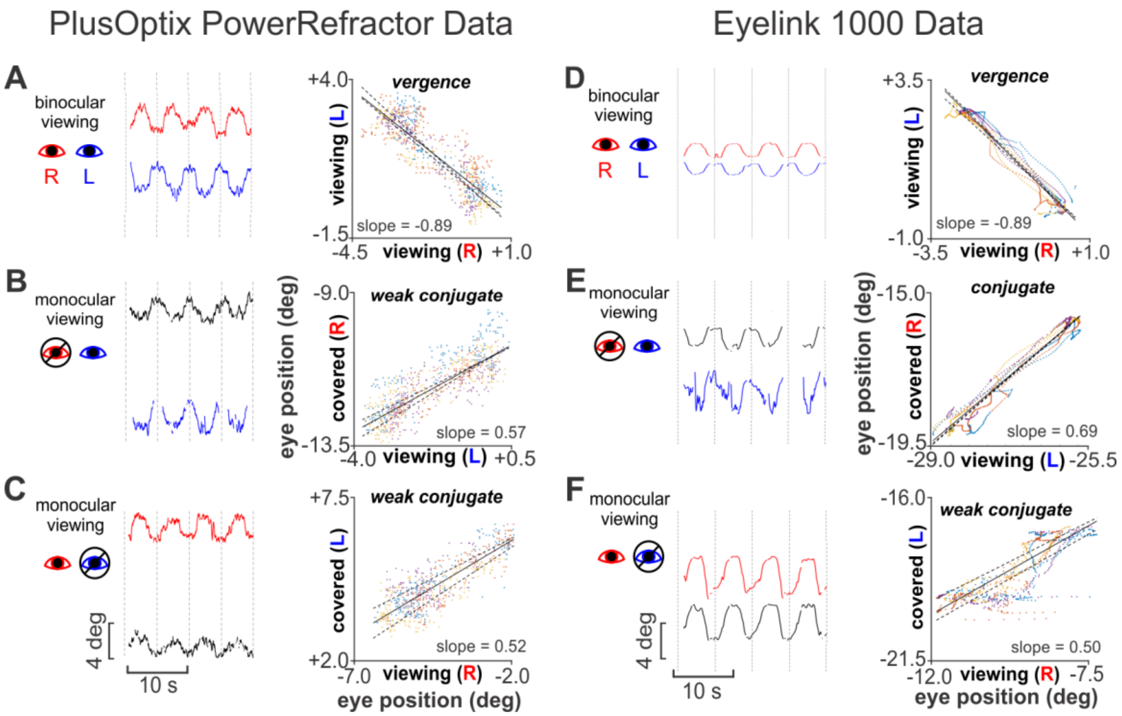

Figure S1. Comparison of PlusOptix and EyeLink data. Eye movements from the same observer (S01) show similar behavior with different eyetrackers. PlusOptix PowerRefractor 3: **A)** Binocular viewing. **B)** Right eye occluded C) Left eye occluded. EyeLink 1000: **D)** Binocular viewing. **E)** Right eye occluded **F)** Left eye occluded. For comparison, EyeLink data was downsampled to 50 Hz. Other details as in Figure 1.

Supplementary Table S1

Subject Number	Age (years)	Corrective Lenses	Visual Acuity (LogMar)		Stereoacuity (TNO)	Cover Test (Diopters)		Fusional Range (Distance)		Fusional Range (Near)	
			OD	OS	seconds of arc	6 meters	33 cms	Base Out (Break/ Recovery)	Base In (Break/ Recovery)	Base Out (Break/ Recovery)	Base In (Break/ Recovery
**1**	42	Glasses	-0.10	-0.20	15	0	2 (xp)	25/20	8/6	40/40	14/12
**2**	26	None	-0.08	-0.08	20	0	4 (xp)	N/A	N/A	N/A	N/A
**3**	22	None	0.00	-0.10	60	0	2 (xp)	8/4	10/6	18/14	12/8
**4**	29	None	-0.30	-0.30	20	0	4 (ep)	N/A	N/A	N/A	N/A
**5**	34	None	-0.10	-0.10	60	0	2 (xp)	16/12	4/2	25/20	12/10
**6**	15	None	0.00	-0.10	60	2(xp)	2 (xp)	N/A	N/A	N/A	N/A
**7**	30	None	0.00	0.00	480	2(ep)	10 (ep)	14/12	10/6	20/18	6/4
**8**	40	None	0.00	-0.20	120	0	0	25/20	6/4	50-50	6-2
**9**	34	None	-0.30	-0.30	30	2(xp)	1(xp)	16/14	6/4	35/30	12/10
**10**	30	None	0.10	0.10	60	0	0	35/30	8/6	40/35	10/8
**11**	30	None	0.20	0.20	60	0	25 (xp)	30/25	12/10	25-20	25-20
**12**	27	None	0.20	0.10	120	2(xp)	8 (xp)	40/35	10/6	25/20	25/20
**13**	29	None	0.10	0.10	N/A	0	0	N/A	N/A	14/10	N/A
**14**	36	None	-0.20	-0.20	60	0	8 (xp)	6/4	8/6	16/14	16/14

Table S1. Participant Clinical Data. 14 participants mean age: 30.29; standard error: 1.86. Visual Acuity was measured with ETDRS Optotype LogMAR Chart with a line-by-line method. Stereoacuity measures were obtained with a TNO Stereotest. For Cover Test and Fusional Range participants fixated the same accommodative target for distance and near measures. Cover Test included Cover-Uncover Test and Alternate Cover Test (ACT). ACT measures reveal latent deviations (phoria) when present within normal range for this population. For Fusional Range; Break Point (BP) refers to participant reporting diplopia (maximum fusional range) and Recovery Point (RP) when participant reported single vision (recovery of fusion); both events were confirmed by a cover test by the tester. RP close to BP indicates good strength of fusional range and control of any latent deviation (phoria). One participant S11 had 25 diopters of well-controlled exophoria for near fixation at 33 cm. and normal stereopsis. ep: esophoria, xp: exophoria (Fusion range was normal in 10 participants. For 4 participants we were unable to measure fusion range; 1 for lack of reliable responses; 3 for logistical reasons; all 4 had typical measures for other parameters)
